# Clinical and Functional Characteristics of a Novel *KLF11* Cys354Phe Variant Involved in Maturity-Onset Diabetes of the Young

**DOI:** 10.1155/2021/7136869

**Published:** 2021-02-01

**Authors:** Yujing Sun, Jingru Qu, Jing Wang, Ruxing Zhao, Chuan Wang, Li Chen, Xinguo Hou

**Affiliations:** ^1^Department of Endocrinology, Qilu Hospital, Cheeloo College of Medicine, Shandong University, Jinan 250012, China; ^2^Institute of Endocrine and Metabolic Diseases of Shandong University, Jinan, 250012 Shandong Province, China; ^3^Jinan Clinical Research Center for Endocrine and Metabolic Diseases, Jinan, 250012 Shandong Province, China

## Abstract

**Background:**

Mutations in human *KLF11* may lead to the development of maturity-onset diabetes of the young 7 (MODY7). This occurs due to impaired insulin synthesis in the pancreas. To date, the clinical and functional characteristics of the novel *KLF11* mutation c.1061G > T have not yet been reported.

**Methods:**

Whole-exon sequencing was used to screen the proband and family members with clinical suspicion of the *KLF11* variant. Luciferase reporter assays were used to investigate whether the *KLF11* variant binds to the insulin promoter. Real-time PCR, western blotting, and glucose-stimulated insulin secretion (GSIS) analysis were used to analyze the *KLF11* variant that regulates insulin expression and insulin secretion activity in beta cell lines. The Freestyle Libre H (Abbott Diabetes Care Ltd) was used to dynamically monitor the proband daily blood glucose levels.

**Results:**

Mutation screening for the whole exon genes identified a heterozygous *KLF11* (c.1061G > T) variant in the proband, her mother, and her maternal grandfather. Cell-based luciferase reporter assays using wild-type and mutant transgenes revealed that the *KLF11* (c.1061G > T) variant had impaired insulin promoter regulation activity. Moreover, this variant was found to impair insulin expression and insulin secretion in pancreatic beta cells. The proband had better blood glucose control without staple food intake (*p* < 0.05).

**Conclusions:**

Herein, for the first time, we report a novel *KLF11* (c.1061G > T) monogenic mutation associated with MODY7. This variant has impaired insulin promoter regulation activity and impairs insulin expression and secretion in pancreatic beta cells. Therefore, administering oral antidiabetic drugs along with dietary intervention may benefit the proband.

## 1. Introduction

*KLF11* was first cloned in a human cystic fibrosis pancreatic adenocarcinoma cell line by Urrutia et al. in 1998 [1]. *KLF11* is a pancreas-enriched transcription factor that has elicited significant attention from researchers due to its role as a negative regulator of exocrine cell growth both *in vitro* and *in vivo* [2]. Previous studies have shown that *KLF11* is involved in the pathophysiological process of diabetes development [3–5]. Moreover, mutations in human *KLF11* may lead to the development of maturity-onset diabetes of the young 7 (MODY7). Neve et al. were the first to study MODY7 [2]. In 2005, genetic screening on two probands with a family history of early onset type 2 diabetes revealed that one proband had type 2 diabetes, while the other had reduced glucose tolerance [2]. This is due to the impairment of insulin synthesis from the pancreas. Genetic analysis of *KLF11* has revealed two rare variants (Ala347Ser and Thr220Met) that are segregated in families with early onset type 2 diabetes and significantly impair its transcriptional activity [2].

In 2019, a novel *KLF11* variant (p.His418Gln) was reported; this was associated with early childhood-onset type 1B diabetes [6]. As such, *KLF11* is a valid candidate gene to determine the genetic predisposition to early onset and type 2 diabetes, as defects in this gene may lead to early onset or polygenic type 2 diabetes [7]. In addition to *KLF11*, there may be other unknown factors that need further exploration. However, *KLF11*, due to its role as a MODY gene, is a potential therapeutic target for adult-onset diabetes.

In this study, we report a novel *KLF11* variant associated with MODY7 and explore its clinical features, possible pathogenesis, optimal treatment, and functional characteristics. Furthermore, we also investigated whether dietary intervention may benefit diabetic patients with *KLF11* mutation.

## 2. Materials and Methods

### 2.1. Patients

#### 2.1.1. Proband

**Figure 1:**
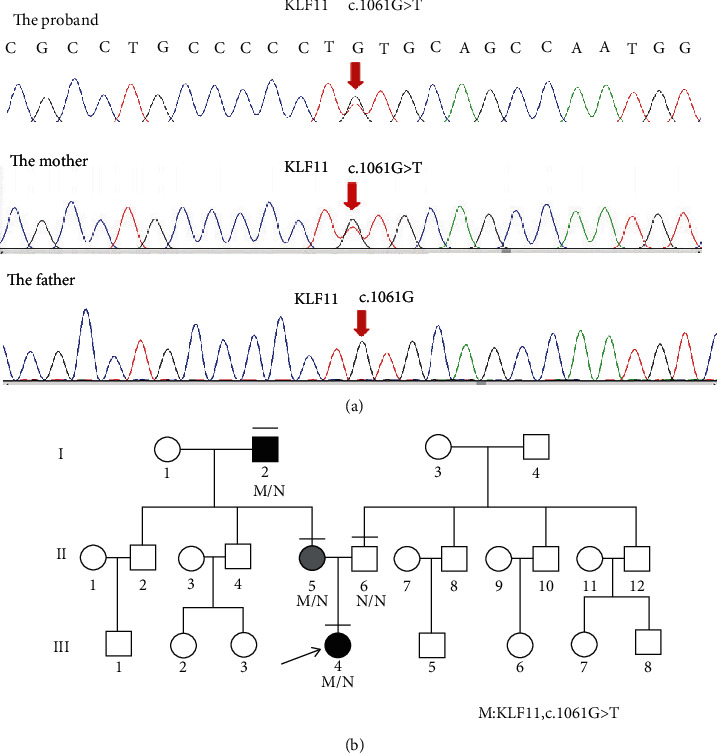
Partial sequence diagram of *KLF11* and the pedigree of the studied family members. (a) Partial sequence diagram of *KLF11*. A heterozygous c.1061G > T transition mutation, causing the substitution of cysteine by phenylalanine at codon 354 is shown using an arrow (GenBank accession number: NM_003597.4). (b) The pedigree of the study family. Women are represented using circles and men, using squares. The black symbols indicate individuals with diabetes. The grey symbol indicates individuals with prediabetes. The proband is denoted by an arrow. The horizontal lines indicate individuals who underwent molecular analysis. The p.Cys354Phe variant of *KLF11* was identified in I-2, II-5, and III-4. The N symbols denote the people that carry the WT gene, and the M symbols denote the people that carry the Cys354Phe variant.

The proband (III-4, Figure 1) was a female subject aged 23 years, with normal development and moderate nutritional status. However, one physical examination on September 2nd, 2018, revealed that the proband's fasting blood glucose level was 13.9 mmol/L. Immediately prior to admission to the local hospital, the proband's fasting blood glucose level was 10.36 mmol/L, and HbA1c levels were 11.5%. Due to the high blood glucose level, the doctor treated her with insulin and oral hypoglycemic agents. One month later, the proband's blood glucose control worsened after gradually adjusting the treatment to saxagliptin (2.5 mg/day) and voglibose (0.6 mg/day), which was followed by admission to the QiLu Hospital for further treatment. The patient reported no polydipsia, polyuria, weight loss, or blurred vision and no numbness of the limbs, fatigue, or discomfort during this time.

All procedures performed in the study involving human participants were in accordance with the ethical standards of the institutional and/or national research committee and with the 1964 Helsinki Declaration and its later amendments or comparable ethical standards. This study was approved by the ethics committee of the QiLu Hospital of Shandong University (No: KYLL-2020(KS)-069). All the subjects included in our study voluntarily signed an informed consent form, which was reviewed by the ethical committee.

#### 2.1.2. Family History

There was no history of diabetes on the paternal side of the family, whereas there was one diabetic patient among maternal relatives (maternal grandfather).

#### 2.1.3. Physical Examination

The proband's height was 168.5 cm; she weighed 54 kg and had a body mass index (BMI) of 19.02 kg/m^2^. Moreover, the proband did not exhibit any obvious abnormalities of the heart, lungs, or abdomen, and there was no edema in either of the lower limbs.

#### 2.1.4. Treatment Methods

Due to the unsatisfactory control of both fasting and postprandial blood glucose levels, the treatment was changed to insulin glargine injections (10 units at bedtime), oral saxagliptin (2.5 mg; once/day), and oral voglibose (0.2 mg; thrice/day).

#### 2.1.5. Clinical Data Collection

The subject's family provided informed consent and was enrolled in September 2018. We collected comprehensive clinical data, such as results of physical examinations, medical history, pedigree, and levels of related metabolism products.

### 2.2. Mutation Analysis

Peripheral blood was collected from four family members (the proband, her parents, and her maternal grandfather). The EDTA anticoagulation and E.Z.N.A.® Blood DNA Mini Kit (omega Bio-Tek, Inc. D3392) were used to extract the genomic DNA from peripheral blood leukocytes, which was then sent to Beijing Fujun Gene Biotechnology Co., Ltd. for whole-exome sequencing.

### 2.3. In Silico Analysis of the KLF11 Variant

Using the phyre2 server, the three-dimensional (3D) structures of wild-type KLF11 (WT-KLF11) and its variant (Cys354Phe-KLF11) were predicted using the threading method and comprehensively analyzed through head-to-head comparison of the final models. The WT-KLF11 and Cys354Phe-KLF11 3D structural models were established as reference models [8]. Structure visualization was performed using the SAVES v5.0 server.

### 2.4. Plasmid Information

The plasmid vector encoding the insulin promoter sequence pGL3-basic-INS, which was first reported by Bernadette Neve et al. [2], was synthesized by Biosune Biotechnology (Shanghai) Co., Ltd. The pCDNA3.1-WT-KLF11 plasmid was purchased from Biosune Biotechnology (Shanghai) Co., Ltd. The pCDNA3.1-C354F-KLF11 plasmid was constructed using the QuikChange site-directed mutagenesis kit (Agilent Technologies, Inc., Santa Clara, CA), according to the manufacturer's instructions. All constructs were verified by sequencing on an ABI 3730xl sequencer. The construct was completely sequenced and used as the template in other cloning designs.

### 2.5. Western Blotting

Human embryonic kidney (HEK) 293 cells were cotransfected with plasmids encoding Cys354Phe-KLF11 and WT-KLF11 and were cultured as previously described [9]. At 48 h posttransfection, the cells were harvested and subjected to sodium dodecyl sulfate-polyacrylamide gel electrophoresis (SDS-PAGE). Western blotting was performed using a mouse monoclonal anti-KLF11 primary antibody (Origene, Cat: TA811001s, 1 : 1000) and horseradish peroxidase-conjugated goat anti-mouse IgG polyclonal secondary antibody (Zhongshan Golden Bridge, Cat: ZB-2305, 1 : 10000). Protein bands were detected using a chemiluminescence kit (Millipore, CA, USA, WBKLS0050) and imaged using a chemiluminescence imaging system (Shanghai Qinxiang Scientific Instrument Co., Ltd.).

### 2.6. Luciferase Reporter Assay

HEK 293 cells were seeded in 96-well culture plates (10,000 cells/well in 200 *μ*L of culture media) and cultured for 24 h. Then, each cell group was transfected separately with a pRL-TK plasmid, pGL3-basic-INS, and pCDNA3.1-WT-KLF11 or pCDNA3.1-C354F-KLF11 using LipofectamineTM 2000(Invitrogen, cat:11668-027), according to the manufacturer's instructions. At 48 h posttransfection, the dual-luciferase reporter assay was performed using the Luciferase Reporter Assay Kit (Promega, E1910).

### 2.7. Insulin Secretion and Insulin Content Assay

INS1 cells were cotransfected with plasmids encoding Cys354Phe-KLF11 and WT-KLF11 to investigate insulin secretion and insulin contents, as described previously [10]. Insulin secretion was determined using a static incubation method under conditions of 5% CO_2_ and 95% air at 37°C, as previously described [11, 12]. Cells were seeded at a density of 2 × 10^5^ cells in 24-well plates and cultured in 1 mL of Dulbecco's Modified Eagle Medium (DMEM, 25 mmol/L glucose). After 48 h, the medium was removed, and cells were washed once with HEPES-balanced Krebs Ringer Bicarbonate Buffer (119 mmol/L NaCl, 4.74 mmol/L KCl, 2.54 mmol/L CaC1_2_, 7.4 mmol/L MgC1_2_, 1.19 mmol/L KHPO_4_, 25 mmol/L NaHCO_3_, 10 mmol/L HEPES, pH 7.4) containing 0.5% bovine serum albumin (BSA) without glucose. Next, the cells were preincubated in HEPES-balanced Krebs-Ringer Bicarbonate Buffer with 0.5% BSA and 5 mmol/L glucose for 30 min. After washing twice with HEPES-balanced Krebs-Ringer Bicarbonate Buffer, INS1 cells were incubated in HEPES-balanced Krebs-Ringer Bicarbonate Buffer supplemented with 0.5% BSA and varying concentrations of glucose. The media were then collected and assayed for immunoreactive insulin via an enzyme-linked immunosorbent assay (ELISA), with mouse insulin being used as a standard. A volume of 200 *μ*L of 1 mol/L NaOH was added to each well to solubilize the cells in order to determine the cellular protein contents using an ELISA assay kit (Millipore, EZRMI-13 K). For the measurement of cellular insulin content, 1 mL of acid ethanol was added to the wells, which were then sealed with a pressure-sensitive film. The extract was collected after 24 h incubation at 4°C and was then diluted and assayed by ELISA.

### 2.8. Real-Time PCR

INS1 cells were isolated using the TRIzol reagent (Takara, T9108). The cDNA was generated using the HiScript Q RT SuperMix for qPCR (Takara, DRR047S), and the real-time PCR assays were conducted with an LC480 Light Cycler using the following primer sequences: Ins1 forward primer, GAAGAGGCCATCAAGCAGATCACT; Ins1 reverse primer, ATTGTTCCACAATGCCACGCT; GAPDH forward primer, GCCTTCCGTGTTCCTACC; and GAPDH reverse primer, GCCTGCTTCACCACCTTC. Relative gene expression was determined using a comparative method (2^-△△CT^). GAPDH was used as an internal standard.

### 2.9. Statistical Analysis

All data are presented as the means ± standard deviations (SDs) or means ± standard errors of the means (SEMs). Statistical comparisons were performed by using two-tailed, paired Student's *t*-test for datasets involving only two groups, or by using one-way ANOVA in the case of data involving more than two groups. Then, the Dunnett's and Bonferroni's post hoc tests were performed for multiple comparisons. All tests were performed using GraphPad Prism 8. Every experiment was repeated at least thrice independently. Representative experimental results are shown in the figures. A *p* value of <0.05 was considered statistically significant.

## 3. Results

### 3.1. Clinical Manifestation

As the proband was first diagnosed with diabetes at the age of 23 and treated with insulin and oral hypoglycemic agents, it was crucial to identify which type of diabetes the proband had. After three weeks of treatment, the HbA1c levels were 6.6%. Therefore, this treatment plan was maintained for one and a half years, and the patient's blood glucose level was controlled and stable during this time.

### 3.2. Laboratory Data

Islet autoantibody screening revealed an absence of glutamic acid decarboxylase (GAD), tyrosine phosphatase antibodies (IA-1ABs), anti-insulin cell antibodies (ICA-IgG), insulin autoantibodies (IAAs), and *β*-cell-specific zinc transporter 8. When the proband's blood glucose level was normal and stable, an oral glucose tolerance test (OGTT) was performed simultaneously with insulin and C-peptide release experiments to assess islet function (Table 1). As the maternal grandfather of the proband was a diabetes patient, we performed genetic testing for the proband. As such, we identified a heterozygous variant of *KLF11* (c.1061G > T, p.Cys354Phe) via whole-exome sequencing. Moreover, the proband exhibited no abnormalities in the thyroid function, hepatic and renal function, blood lipid profile, or urine microalbumin.

### 3.3. Clinical Characteristics of Family Members and Their Genetic Testing Results

The maternal grandfather of the proband (I-2) was diagnosed with type 2 diabetes at 66 years of age and treated with oral hypoglycemic agents. His blood HbA1c level was 6.0% during his last examination (Table 2). The parents of the proband were never diagnosed with diabetes, but the mother (II-5) was found to have a fasting blood glucose level of 5.8 mmol/L at the physical examination. Genetic testing for MODY facilitates a correct diagnosis, thereby enabling treatment optimization and allowing the monitoring of asymptomatic family members. Therefore, genetic tests were conducted for the parents and maternal grandfather of the proband. As expected, the heterozygous variant of *KLF11* (c.1061G > T, p.Cys354Phe) was also identified in the mother and maternal grandfather (Figure 1(a)). In order to confirm whether the mother was an asymptomatic member, we performed an OGTT simultaneously with insulin and C-peptide release experiments (Table 1). Our results showed that the mother of the proband can be diagnosed with impaired glucose tolerance, which means she is prediabetic and may develop diabetes in the future.

As there are differences in the clinical phenotypes caused by the same mutation even within the same family, these results may indicate that *KLF11* mutations are associated with incomplete penetrance. In summary, our results indicate that the *KLF11* (c.1061G > T) variant is associated with diabetes in this family.

### 3.4. Sequencing Results and Biochemical Characterization of the KLF11 (c.1061G > T) Variant

**Figure 2:**
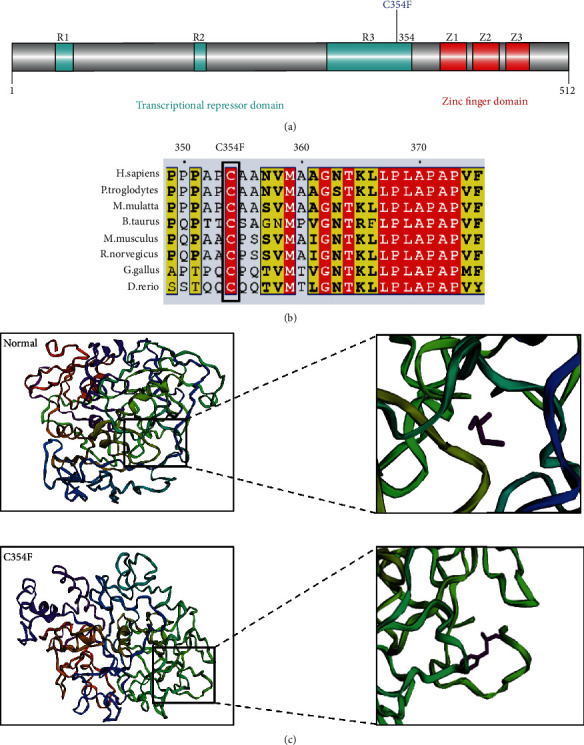
Sequencing analysis and 3D structure of the mutant protein. (a) KLF11 structure domains. Mutations at the protein level are indicated below the 3RDB domain. (b) Cross-species conservation of Cys354Phe-KLF11. (c) Protein structure prediction of KLF11 (WT and Cys354Phe).

The conserved domain of human *KLF11* consists of an extracellular region that comprises three transcriptional repressor domains (TRD) and a zinc finger domain. *KLF11*, as a member of the Sp1/KLF family, has been predicted to bind to either GC-rich or CACC sequences. The Cys354Phe-KLF11 variant was mapped to a novel hydrophobic glycine-glutamine-proline-rich domain that was observed in the corresponding region of its fly ortholog, cabut (Figure 2(a)). The KLF11 Cys354Phe variant was also mapped to the previously characterized transcriptional regulatory domain 3 (TRD3). Multiple amino acid sequence alignments using Clustal W showed that Cys354Phe-KLF11 was conserved across various species (Figure 2(b)). It was predicted that mutations in this buried site led to its exposure on the surface of the protein, thereby modifying the protein activity. In addition, due to the exposure of this site at the surface of the protein after mutation, it might be located in a larger transcriptional blocking domain and affect some transcriptional functions (Figure 2(c)).

### 3.5. Functional Characterization of the KLF11 (c.1061G > T) Variant

**Figure 3:**
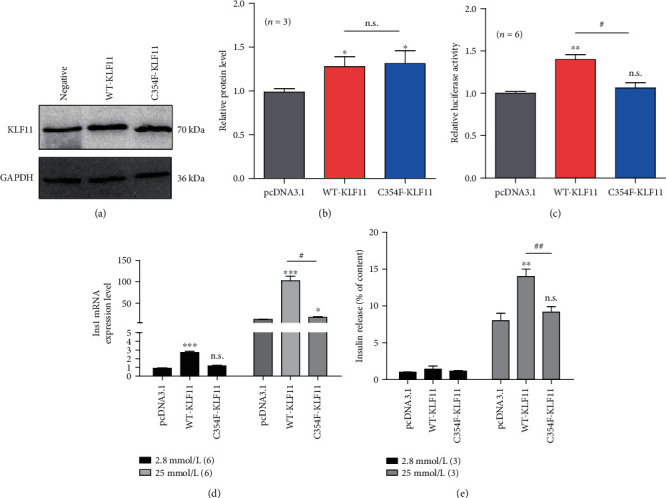
Functional analysis of the KLF11 Cys354Phe variant. (a) Protein expression of KLF11 (WT and Cys354Phe). Cell lysates of KLF11-expressing cells were used. Protein production was tested via western blotting. (b) Histogram of the KLF11 protein expression level analysis. (c) Luciferase assays of HEK 293 cells transfected with each KLF11 expression vector (WT and Cys354Phe). ^∗∗^ denotes *p* < 0.01 (*p* = 0.0031) for the KLF11 WT plasmid compared to the empty plasmid; n.s. denotes *p* > 0.05 (*p* = 0.3899) for KFL11/C354F compared to the empty plasmid; # denotes *p* < 0.05 (*p* = 0.0176) for KFL11/C354F compared to KLF11 WT. (d–f) INS1 cells were transfected with the KLF11-WT or KLF11-Cys354Phe plasmids for 24 (d) or 48 h (e). (d) qRT-PCR was conducted to determine the Ins1 mRNA levels after stimulation with 2.8 mmol/L glucose (low) or 20 mmol/L glucose (high) for 24 h. (e) Insulin secretion levels in INS1 cells were analyzed via glucose-stimulated insulin secretion assay using ELISA after stimulation with 2.8 mmol/L glucose (low) or 20 mmol/L glucose (high) for 2 h. Note: *n* denotes the number of experiments. ^∗∗^ denotes *p* < 0.01 (*p* = 0.0018) for KLF11 WT compared to the empty plasmid; n.s. denotes *p* > 0.05 (*p* = 0.1836) for KFL11/C354F compared to the empty plasmid; ## denotes *p* < 0.01 (*p* = 0.0027) for KFL11/C354F compared to KLF11 WT.

The link between the *KLF11* (c.1061G > T) variant and the putative diabetes pathophysiological process was assessed. The *KLF11* (c.1061G > T) gene variant did not affect protein expression levels (Figures 3(a) and 3(b)). The results of the luciferase assays demonstrated that *KLF11* plays a role in and the transcriptional regulation of the insulin gene. The insulin promoter activity induced by the *KLF11* (c.1061G > T) variant was observed to be lower than that induced by WT-KLF11 (Figure 3(c)). Moreover, the insulin gene activation was observed to be affected in the mutant *KLF11*. To further explore the role of the *KLF11* Cys354Phe variant on *β*-cell function, we overexpressed the WT and variant *KLF11* in INS1 cells (using the pcDNA3.1 plasmid as the negative control and the *KLF11* (WT and Cys354Phe) plasmids) and found that the *KLF11* Cys354Phe variant decreased insulin transcription and reduced insulin secretion even after stimulation with high glucose (Figures 3(d) and 3(e)).

### 3.6. Exploration of KLF11 (c.1061G > T) Gene Variant Treatment

**Figure 4:**
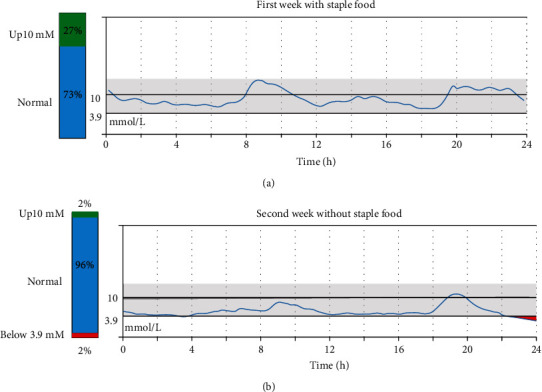
Representative data of the scanning dynamic glucose monitoring report for the proband with and without staple food intake. The proband wore the Freestyle Libre H (Abbott Diabetes Care Ltd.) for the dynamic monitoring of the blood glucose levels for 14 days. The proband needed to maintain normal staple food intake during the first week, while no staple food intake was allowed during the second week; the amount of calories for each meal was recorded through the “mint app” (China) on the phone. No exercise adjustments were performed in these two weeks. After 14 days, the data were analyzed to obtain the following results: (a) representative data of the scanning dynamic glucose monitoring report for the proband with staple food intake. The proband was shown to have a glucose level above 10 mmol/L for approximately 27% of the day; (b) representative data of the scanning dynamic glucose monitoring report for the proband without staple food intake. The proband was shown to have glucose levels above 10 mmol/L for only about 2% of the day.

The blood glucose level of the proband was under control after treatment with insulin and oral hypoglycemic agents for one and a half years (Table 2). Due to the inconvenience of using insulin at school, the medication was adjusted to oral gliclazide sustained-release tablets (30 mg/day) combined with diet control in June 2020. We found that together with the control of staple food intake, this treatment resulted in better blood sugar control in a two-week staple diet trial. There was no significant difference in energy intake between the two weeks (*p* < 0.05, Table 3). However, there were significant differences in average daily blood glucose, fasting blood glucose, and 2 h postmeal blood glucose between the two weeks (*p* < 0.05, Table 3). There was no statistical difference in the mean amplitude of glycemic excursions (MAGE) between the two weeks, which represented blood glucose fluctuations. According to the dynamic blood glucose parameters, dietary intervention may be beneficial for the proband and help control daily glucose levels (Figures 4(a) and 4(b)).

## 4. Discussion

The diagnosis of MODY is challenging due to its relatively low prevalence and the overlap in presentation and clinical features between patients with MODY and those with other diabetes subtypes [13]. MODY is characterized by autosomal-dominant inheritance with a multigenerational family history of diabetes, onset before 25 years of age, and the absence of pancreatic autoimmunity and insulin resistance [14]. In this study, we identified a *KLF11* variant in three individuals belonging to one family via whole-exome sequencing. Among them, two individuals (including one young adult) developed diabetes. The proband exhibited hyperglycemia at 23 years of age and was observed to be negative for islet cell autoantibodies. The maternal grandfather developed type 2 diabetes at the age of 66. The mother was considered non-diabetic,but had delayed insulin secretion via OGTT and islet function test, which is one of the characteristics of diabetes. The father was considered healthy. In fact, strict adherence to these guidelines confers high specificity but low sensitivity in identifying MODY subjects, as more than half of patients with confirmed mutations identified in European countries do not meet these clinical criteria for referral. As such, adherence to current guidelines will continue to lead to the misdiagnosis of a large proportion of patients with MODY [15, 16]. Furthermore, by extending MODY diagnostic testing beyond current guidelines, Owen et al. [17] identified MODY subjects with clinical features that are not characteristic of MODY, including the absence of a family history of diabetes. Therefore, the diagnosis of MODY for the proband is definite, even without a typical family history.

It is well known that diabetes is the result of a combination of genetic and environmental risk factors. Among them, epigenetics plays a vital role in mediating the interaction between environmental and genetic factors. Epigenetics also has an intergenerational effect, that is, some extragene information from the grandparents will be passed to the grandchildren, and marks will be burned on the grandchildren, who show the corresponding characteristics. These factors may contribute to the earlier onset of diabetes.

Genetic studies have found that missense mutations in the *KLF11* gene lead to the development of late-onset diabetes [7]. This is due to the fact that the mutations affect *KLF11*-binding promoters and activation of the bladder protein gene. Moreover, after mutations in the cis-acting element of *KLF11* were found to inhibit *KLF11*-induced activation of the insulin gene, leading to a decrease in the biosynthesis of insulin in the body. The KLF11 protein is a zinc finger transcription factor that binds to SP1-like sequences in the promoter region of the *ε*- and *γ*-globin genes [18]. Three transcript variants encoding two different isoforms have been found for *KLF11* mRNA, and their proteins are expressed in pancreatic *β*-cells [10]. This binding increases its repression and impairs the activation of insulin promoters [19]. In addition, defects in this gene cause MODY7. Furthermore, Neve et al. identified two rare *KLF11* variants (Ala347Ser and Thr220Met) in families with early onset type 2 diabetes, which were shown to significantly impair the transcriptional activity of *KLF11*. Furthermore, they discovered a frequent polymorphic Gln62Arg variant that was significantly associated with type 2 diabetes mellitus in North European populations [2]. Ushijima et al. identified a heterozygous *KLF11* (p.His418Gln) variant in a family that was clinically diagnosed with early childhood-onset type 1B diabetes [6]. These two diabetes-associated studies revealed the effects of a loss of *KLF11* protein function.

Multiple amino acid sequence alignments showed that KLF11 Cys354Phe (C354F) was conserved across various species. Moreover, the cells transfected with the *KLF11*-WT and mutant plasmid constructs exhibited no statistically significant differences (data not shown). Moreover, there were no significant differences in the protein expression levels between the *KLF11-*WT- and *KLF11*-C354F-transfected cells. Notably, the *KLF11* (c.1061G > T) variant induced lower insulin promoter activity than *KLF11*-WT. This indicates that the mutant affects the *KLF11*-induced activation of the insulin gene. It is predicted that mutations at this buried site may lead to its exposure on the surface of the protein, thereby altering the protein activity [20, 21]. In addition, due to the exposure of this site at the surface of the protein after mutation, it might be possible that the site is located in a larger transcriptional blocking domain, thereby affecting the transcriptional functions of insulin [22]. Further cell function studies showed that the *KLF11* Cys354Phe variant decreased insulin transcription and reduced insulin secretion even upon stimulation with high glucose. Moreover, dysregulation of *KLF11* also affected the insulin content of cells.

Children and adolescents diagnosed with diabetes may initially be treated with insulin, and this regimen often continues even after the stabilization of glycemia. However, in some patients with MODY, hyperglycemia can be controlled by prescribing oral antidiabetic drugs (e.g., sulfonylureas), without the use of insulin [23]. In fact, standard treatments have not been established for most diabetes subtypes due to the low number of cases and lack of data confirming treatment efficacy. Administration of insulin therapy from the initial phase of MODY7 has been described in previous study [6], which is consistent with the treatment received by the proband in our study. In this case, using insulin (using and storage defects) also led to inconvenience for the proband. Moreover, after treatment with sulfonylureas and when the total energy intake remained stable by reducing the intake of staple foods, we found that the proband's blood glucose levels were better controlled. Therefore, oral antidiabetic drugs and dietary intervention may benefit diabetic patients with *KLF11* mutations and help them control their daily glucose levels. The following regimen and a healthy lifestyle are very important factors in the disease control for patients with type 2 diabetes.

## 5. Conclusions

In summary, in this study, we successfully identified the *KLF11* (c.1061G > T) variant via whole-exome sequencing, which was shown to cause MODY7 in a 23-year-old female. This study is the first to demonstrate that *KLF11* (c.1061G > T) variants are involved in the pathogenesis of MODY7. Epigenetic factors may contribute to the earlier onset of diabetes. Moreover, we showed that the administration of oral antidiabetic drugs and dietary interventions were beneficial for the proband and helped control the daily glucose levels. Our study also has a few limitations. Further studies, such as animal experiments, are needed to explain the association between altered *KLF11* function and the diabetes pathogenesis and severity. In addition, new dietary intervention and treatment methods need to be developed for MODY.

## Figures and Tables

**Table 1:** Blood glucose, insulin, and C-peptide levels of the proband and her mother.

Time (h)	Blood glucose (mmol/L)		Insulin (*μ*IU/mL)		C-peptide (ng/mL)	
	II-5	III-4	II-5	III-4	II-5	III-4
0	5.27	6.98	3.12	8.99	0.66	0.31
0.5 h	10.47	9.02	24.1	11.27	3.97	0.54
1 h	10.91	10.3	33.38	15.1	5.63	0.95
2 h	6.57	12.43	59.63	17.45	6.37	1.44
3 h	3.38	9.81	29.38	15.2	3.07	1.49

**Table 2:** Clinical characteristics of the study family.

	Proband	Maternal grandfather	Mother‡	Father‡
Birth weight (kg)	3.15	No data	No data	No data
At diagnosis				
Age (yr)	23	66	—	—
Height (cm)	168.5	172	—	—
Body weight (kg)	54	65	—	—
BMI (kg/m^2^)	19.02	21.97	—	—
DKA†	No	No	—	—
Blood glucose (mmol/L)	13.9	No data	—	—
HbA1c (NGSP, %)	11.5	No data	—	—
At the latest examination				
Age (yr)	24	70	50	50
Height (cm)	168.5	172	163	173
Body weight (kg)	50	55	50	65
BMI (kg/m^2^)	17.61	18.59	18.82	21.72
HbA1c (NGSP, %)	5.9	6.0	5.1	4.6
Fasting serum C-peptide (ng/mL)	0.31	0.72	0.50	0.73
Fasting serum insulin (*μ*IU/mL)	8.99	4.74	4.09	2.65
Treatment plan	Insulin glargine injection (10 units at bedtime), saxagliptin (2.5 mg once/day), voglibose (0.2 mg three times/day)	Metformin hydrochloride 1000 mg/day; gliclazide 160 mg/day	—	—

†DKA: diabetic ketoacidosis. ‡This individual had no diabetes.

**Table 3:** The mean and standard deviation of blood glucose and daily energy intake of the proband between the two weeks.

Variable		First week with staple foods		Second week without staple foods		*p* value
		Mean	SD	Mean	SD	
Blood glucose (mmol/L)	Daily average	5.65	0.49	4.16	0.54	0.000
	Fasting	5.21	0.52	4.09	0.78	0.01
	2 h after breakfast	7.03	0.38	5.15	1.14	0.004
	2 h after lunch	5.8	1.13	4.2	1.20	0.054
	2 h after dinner	7.12	1.91	4.87	0.86	0.021
Mean amplitude of glycemic excursions (MAGE)		2.88	0.83	2.93	0.87	0.918
Daily energy intake (kilocalories)		918.28	287.90	1023.00	507.61	0.643

## Data Availability

The data used to support the findings of this study are available from the corresponding author upon request.
